# Causes of Albuminous Urine

**Published:** 1852-03

**Authors:** 


*Causes of Albuminous Urine.*—M. Ed. Robin lately read a paper on the above subject before the Academy of Medicine of Paris; we subjoin an abstract of the same:—In the normal state the albumen is burnt in the blood, and the nitrogenized residue of this combustion—viz., urea and uric acid—is eliminated by the urine. The combustion is, however, not so complete as not to allow some little albumen to escape with the renal secretion; but this albumen, besides being very small in amount, is somewhat different from the ordinary kind. M. Robin thinks that if during a sufficiently long time the albumen underwent in the circulation a much smaller amount of combustion than is habitually the case, it might pass unaltered into the urine, instead of being thrown off in the form of urea and uric acid. The author cites the following facts in support of his opinion:—

The urine becomes albuminous in croup, in complete ascites, and in cases of capillary bronchitis, with emphysema, accompanied by much dyspnœa; in pulmonary phthisis, especially when complicated by pneumonia and marked with difficult breathing; in gestation, when sufficiently advanced to occasion an habitual congestion of the kidneys, owing to an impeded abdominal circulation; and in such states of the system in which a very incomplete respiration causes a marked diminution of combustion. The urine is also albuminous in cyanosis, of whichever nature it may be; in affections of the heart when they exist in such a degree as to keep the patients in a state of semi-asphyxia; and, of course, in such cases where an obstacle to the circulation of the blood, or a malformation of the heart, prevents the hæmatosis from being as rapid as under ordinary circumstances. The urine is likewise albuminous in idiopathic or traumatic lesions of the nervous centres, which cause a lowering of temperature, and thereby a marked decrease of combustion; in diabetes, a disease where very often a lesion of the nervous centre seems to be the origo mali; where the great abundance of sugar in the blood seems to be an obstacle to the combustion of albumen; and where finally the natural heat is lowered by one or two degrees with patients who are severely affected. The urine is albuminous in that kind of nervous exhaustion which characterizes the state of frame called lumbago, which exhaustion must be connected with a great diminution of calorification, and slow combustion. The urine is likewise albuminous in consequence of severe exposure to cold of a large surface of the body. Finally, Bright's disease, where the urine is always albuminous and anæmic, is especially attributed to many of the causes which have been above enumerated as capable of exciting the passage of albumen into the urine.

The author continues by stating that some useful data may be obtained from comparative physiology. As a general rule the urine of the common mammalia and of birds contains no albumen. Among reptiles, on the other hand, the batrachia, so remarkable by the low temperature of their animal heat, yield urine in which albumen is always to be found. It now remains to be proved, says M. Robin, that the urine becomes albuminous under the influence of such agents as interfere in a marked degree with slow combustion. The author then adduces the following conclusions :—

When the activity of the combustion which takes place in the blood is too feeble to burn the whole of the albumen which, in the normal state, should be consumed in a given time, the general vitality is diminished, and thus more or less albumen is allowed to pass unaltered into the urine,—viz., just so much organic matter as escapes the transformation into urea or uric acid. The proportion of urea contained in albuminous urine should therefore be smaller than it is found in normal urine, and such is found to be the case in the following diseases, the only ones, according to the author, in which experiments have been made—viz., pulmonary phthisis, diseases of cerebro-spinal axis, extensive and acute bronchitis with intense dyspnœa, and Bright's disease.—*London Lancet.*

